# “Extended Descriptive Risk-Averse Bayesian Model” a More Comprehensive Approach in Simulating Complex Biological Motion Perception

**DOI:** 10.3390/biomimetics9010027

**Published:** 2024-01-03

**Authors:** Khashayar Misaghian, J. Eduardo Lugo, Jocelyn Faubert

**Affiliations:** 1 Sage-Sentinel Smart Solutions, 1919-1 Tancha, Onna-son, Kunigami-gun, Okinawa 904-0495, Japan; jocelyn.faubert@umontreal.ca; 2 Faubert Lab, School of Optometry, Université de Montréal, C.P. 6128, Montreal, QC H3C 3J7, Canada; 3 Facultad de Ciencias Físico-Matemáticas, Benemérita Universidad Autónoma de Puebla, Av. San Claudio y Av. 18 Sur, Colonia San Manuel Ciudad Universitaria, Puebla Pue 72570, Mexico

**Keywords:** biological motion, Bayesian, dorsal pathway, hierarchical simulation model, reaction time

## Abstract

The ability to perceive biological motion is crucial for human survival, social interactions, and communication. Over the years, researchers have studied the mechanisms and neurobiological substrates that enable this ability. In a previous study, we proposed a descriptive Bayesian simulation model to represent the dorsal pathway of the visual system, which processes motion information. The model was inspired by recent studies that questioned the impact of dynamic form cues in biological motion perception and was trained to distinguish the direction of a soccer ball from a set of complex biological motion soccer-kick stimuli. However, the model was unable to simulate the reaction times of the athletes in a credible manner, and a few subjects could not be simulated. In this current work, we implemented a novel disremembering strategy to incorporate neural adaptation at the decision-making level, which improved the model’s ability to simulate the athletes’ reaction times. We also introduced receptive fields to detect rotational optic flow patterns not considered in the previous model to simulate a new subject and improve the correlation between the simulation and experimental data. The findings suggest that rotational optic flow plays a critical role in the decision-making process and sheds light on how different individuals perform at different levels. The correlation analysis of human versus simulation data shows a significant, almost perfect correlation between experimental and simulated angular thresholds and slopes, respectively. The analysis also reveals a strong relation between the average reaction times of the athletes and the simulations.

## 1. Introduction

The consistent flowing movements that are unique to biological agents are known as the biological motion phenomenon. Biological motion can reveal a variety of information, such as the identity of a predator or the intent of a person in a social context. Accurately perceiving these cues, even under suboptimal conditions, can ensure an observer’s physiological or social survival [1,2]. Biological motion can be efficiently recognized from point-light displays, which are a number of lit points representing significant joints of the biological agent [2,3]. While the body of research in this domain is vast and diverse, it mainly focuses on three prominent themes: kinematic information captured by the visual system, underlying neural mechanisms of biological motion perception [1,4], and the brain’s sensory and motor areas’ participation in biological motion perception [5].

Due to the massive number of imaging, neurophysiological, and psychophysical studies on the subject, there appears to be unanimous agreement on the major activated areas of the brain during the biological motion perception process. The dorsal and ventral pathways of the visual system, along with the site of their convergence in the superior temporal sulcus (STS), are considered to be the primary activated areas during the process [6,7]. 

Naturally, over time, the accumulation of data from biological motion experiments and the need for a sound theoretical framework have necessitated a computational model that could explain biological motion perception in a neurophysiologically plausible fashion [1]. Therefore, based on the idea of the formation and presence of saved prototypical patterns in the visual system, Giese et al. [4] proposed a feed-forward multi-level architecture with one stream for dynamic form detection and one stream to detect the optic flow, simulating the dorsal and ventral pathways of the visual system. Using this simulation model in their study, they concluded that it is the dorsal pathway (motion analysis stream) that has the most impact on biological motion perception [1,4,8]. This finding was at variance with the earlier study on the matter by Beintema and Lappe [6]. Later, it was argued that the perception/recognition of biological motion requires synergy between two pathways to achieve the most robust response [1].

Meanwhile, developmental studies reveal an exclusive predisposition towards motions emanating from biological agents in human infants and newborn fowls, while no exclusivity has been distinguished about organic shapes and forms over non-biological ones [9,10]. Also, behavioral data indicate the existence of some low-level motion capturing filters specialized in biological motion [11,12]. Consequently, Thurman and Lu [13] showed that the ventral pathway processes the biological and non-biological dynamic forms similarly, challenging the existence of any substrate specialized in processing dynamic biological form exclusively. 

Although further examinations of the neural correlates show the activation of some cortical regions linked to form and motion cues in addition to the ventral and dorsal pathways in the event of biological motion perception, the nature of those activations could not be appraised as causal in nature [3,14,15]. Consequently, one study investigated six patients with compromised visual cortex with focal injuries in multiple regions of their ventral pathway. Accordingly, all subjects were capable of recognizing the point-light stimuli while maintaining not significantly different thresholds compared to intact subjects. Surprisingly, they exceeded other patients who had injuries in other areas of the brain critical to biological motion perception [3]. 

To explore the potentials and limits of the dorsal pathway, considering the connotations from all recent findings and the apparent need to address biological motion perception in a more realistic context, the necessity of a more effective biologically plausible computational framework appears desirable [16,17]. 

In a more pragmatic approach, Misaghian et al. [16]. proposed a biologically plausible simulation model that could anticipate the direction of the ball from a wide range of biological motion stimuli of a soccer player, adopted from Romeas et al. [18], while previous approaches showed no promise when confronted with these stimuli. Based on the assumption that the visual system recognizes and makes a decision using stored prototypical patterns [4], our model managed to replicate the psychometric functions of 11 athlete subjects from a behavioral study accurately [16,17,18]. The model consists of hierarchies parallel to the motion pathway hierarchy levels in the visual system [4,8] while incorporating a descriptive risk-averse Bayesian scheme for pattern recognition and a more robust version of a mutual inhibition network for decision making [16,17]. Despite the model’s success, it could not contain all the subjects’ performances from the psychophysical study. 

## 2. Model

**Figure 1:**
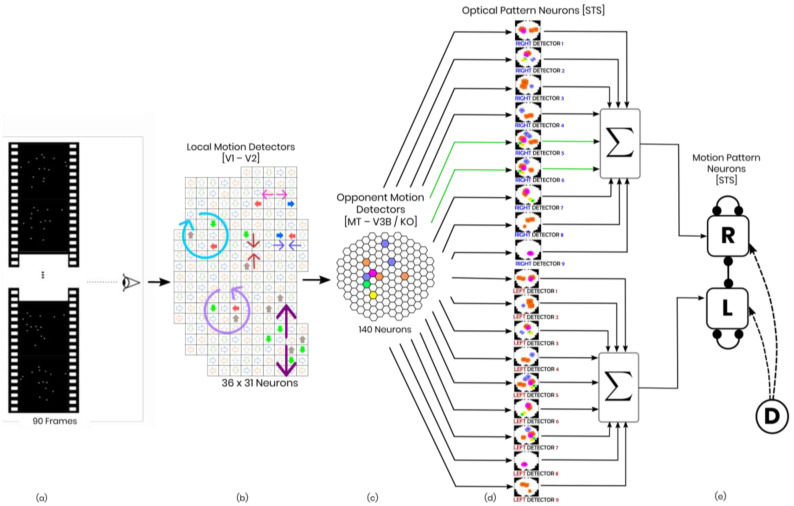
Schematic of the model in one hypothetical point in time, from left to right: (**a**) the reel of biological motion stimulus; (**b**) local motion detectors as ensemble of 1116 neurons positioned in a 36 by 31 arrangement, firing due to the motions they have experienced during two consecutive frames, are represented by the cells with color−filled arrows (blue: right, orange: left, grey: up, green: down); the larger, two−headed or curved, colorful arrows were drawn to display the types of opponent motions that would be sensed on the next level (cyan: horizontal expansion, orange: vertical expansion, magenta: vertical contraction, green: counter−clockwise rotation, and yellow: clockwise rotation); (**c**) opponent motion detectors, the ensemble of 140 neurons to detect horizontal expansion, horizontal contraction, vertical expansion, and vertical contraction, the activated detectors are marked with color−filled hexagons with their corresponding color (cyan: horizontal expansion, orange: vertical expansion, magenta: vertical contraction, green: counter−clockwise rotation, and yellow: clockwise rotation); (**d**) optical−flow pattern detectors, an arrangement of 18 neurons following a one−dimensional mean−field dynamics, each neuron incorporates a statistical template (displayed as colorful map) that represents a specific part of the manifold of the kicking sequences (for example neuron number 2 contains a template for the seconds 11 to 20 of the kick−to−right sequence, while neuron number 10 would have a larger instantaneous input for the seconds 1 to 10 of the kick to the left stimulus). Green arrows highlight the contribution of two cells to the evidence integration at that hypothetical point due to the similarity of the evidence signal and their template (**e**) thresholding stage, two decision neurons for the right and left decisions (marked by capital letters R and L on the square cells with soft edges) are follow our mutual inhibition dynamics, receiving their corresponding inputs from integration stage, and the straight and curve lines with rounded heads highlight the inhibitory interaction between the neurons and the auto-inhibition, respectively. No activity could be seen by either of the neurons since at that hypothetical point in time, neither had made a decision yet. Also, the dotted curved arrows and the circle with the letter D represent the disremembering mechanism.

The feed-forward Bayesian model mentioned earlier is a risk-averse model that consists of hierarchies of neurons representing the dorsal stream. The model consists of three levels of neurons that detect different motion patterns (Figure 1).

### 2.1. Local Motion Energy Detectors

At the first level, there are local motion energy detectors, which are sensitive to small receptive fields with motion directions (≈0.4 degrees). The model incorporates sensitivity to four relative directions in 2D (up/down/left/right). The receptive fields are arranged in a 36 × 31 grid with some overlapping [19]. The activity of local motion detectors is approximated using the vector fields obtained from calculating optical flow in all consecutive frames of the stimulus. This level of neurons has been reported in areas V1/2 and area MT of the monkey’s visual cortex.

### 2.2. Opponent-Motion Detectors

The second level of the neural hierarchy is concerned with neurons that respond to expansions, contractions, and rotations happening in the nearby subfields of their receptive fields [20]. Within each subfield, these detectors combine (max-pools) the responses of the local energy neurons from the lower level to create a signal. This signal is a product of the maxima and suggests spatial invariance within the receptive field [21,22,23,24]. The human brain’s KO/V3B region is believed to have neurons that detect opponent motion [25,26].

To gain insight into the role of clockwise and counterclockwise rotation detectors in biological motion perception, they are added to the previous horizontal and vertical contraction and expansion sensors [16,17]. The new arrangement includes two groups of 5 × 4 assemblies that account for clockwise and counterclockwise detectors, respectively. In total, there are 20 new rotation detectors, bringing the total number of detectors to 100 [8,16,17].

### 2.3. Complex Global Optic Flow Pattern Detectors

The next hierarchy-level detectors have receptive fields covering the whole stimulus (>0.8 degrees) and recognize complex optic flow patterns in the right temporal order. These detectors are specifically tuned to a particular optic flow pattern of a certain temporal order. To simulate the third hierarchy level, we have maintained the same structure as the previous study [16,17]. 

Our model has 18 neurons at this level that are asymmetrically and laterally connected. These connections are arranged so that the active neuron excites the neurons tuned to the future templates and inhibits the ones tuned to the past [4,8,27].

The dynamic of a detector sensitive to the optic flow pattern in the ith frame (the optic flow risen from the i−1 to *i*-th frame) of one stimulus sequence is as follows [8]:

(1) $$\tau_{OFP}={\dot{H}}_{i}\left(t\right)+G_{i}\left(t\right)+\sum_{m}w\left(i-m\right)f\left(H_{i}\left(t\right)\right)$$

where $H_{i}\left(t\right)$ is the activity of the detector i, $\tau_{OFP}=150$ ms and is also the time constant of the global optic flow pattern layer dynamic, w(m) is a weight kernel, f(H) is a step threshold function, and the instantaneous template matching performed by the neuron is modeled as the feed-forward input $G_{i}\left(t\right)$. Here, our previously designed descriptive risk-averse Bayesian classifier generates the feed-forward input. Refer to Misaghian et al. [16] for a thorough description of this approach.

Different areas in the superior temporal sulcus have been known to be the most probable locations of the complex optic flow pattern neurons [28,29,30,31].

### 2.4. Complete Biological Motion Pattern Detectors (Motion Pattern Detectors)

The robust mutual inhibition model proposed by Misaghian et al. [16] could not explain the reaction time of the athlete subjects in Romeas and Faubert’s study [18] as it lacked a mechanism for it. Therefore, we introduced a disremembering strategy to the model, which enabled motion pattern neurons to make consistent decisions within a similar reaction time frame. 

Neural adaptation is a phenomenon in which neurons gradually become less responsive to a constant stimulus over time. This has been observed in the visual system, where the perception of an image or motion diminishes or disappears if there is no micro-saccadic eye movement [32]. Adaptation phenomena have also been studied in the context of decision-making [33,34], including complete motion pattern neurons, which cannot remain activated indefinitely. Our disremembering strategy accounts for the fleeting nature of neural activation in our model.

### 2.5. Robust Mutual Inhibition Model with Adaptation

The dynamic below explains the response of decision-making neurons in the mutual inhibition model [35].

(2) $$\begin{array}{c} \tau \frac{dT}{dt}=-T+S\left(P_{T}\left(D\right)\right),\\ \tau \frac{dD}{dt}=-D+S\left(P_{D}\left(D,T\right)\right), \end{array}$$

where, T is the activity of a primarily excited neuron and D represents the activity of other neurons. τ is a time constant and S() is a modified Michaelis–Menten function [36], which has been proven advantageous in excitatory–inhibitory network model design. Also, $P_{T}$ and $P_{D}$ are the information thresholds [35]. When information thresholds are negative and, as a result, the neurons lateral connections are off, the robust mutual inhibition model sets the negative output of the neurons into zero. For more information on the mechanism of robust mutual inhibition, one must refer to Misaghian et al. [16].

To actualize the neural adaptation in our model, we add the following terms as an input to the dynamic of both T and D neurons:

(3) $$\begin{array}{c} D_{iS_{T}}=u\left(t-\tau_{a}\right)*\left[T-S\left(P_{T}\left(D\right)\right)-kT\right],\\ D_{iS_{D}}=u\left(t-\tau_{a}\right)*\left[D-S\left(P_{D}\left(D,T\right)\right)-kD\right], \end{array}$$

where, u() is the unit step function, $\tau_{a}$ marks the time point when adaptation starts, and k is just a weighting coefficient. As one can see, at $\tau_{a}$, the disremembering inputs to the differential equations become switched on, and the dynamic of each neuron reduces to a simple exponential decline, driving the neuron out of the excitation state.

### 2.6. Modeling Internal Noise

Like our previous study, to model the uncertainty, the output of each optic flow pattern neuron is considered to be elicited from $N\left(H_{i}\left(t\right),\Delta t\delta^{2}\right)$$N(H_{i}(t),∆t\delta^{2})$, where N() is a Gaussian process with the mean, $H_{i}\left(t\right)$ is the activity of the optic flow neuron i without noise, and $\delta^{2}$ is the variance of the added internal noise [16,17].

## 3. Methods

The simulation model was developed using Matlab, while the data and statistical analyses were performed in the R Studio framework. To maintain similarity with human subjects, the original point-light soccer kick from Romeas and Faubert’s study [18] was adopted. The stimulus was a 90-frame animation lasting 4.5 s and showed bright point-lights representing significant joints of the human body against a dark background. The leftward and rightward kicks were synthesized by rotating the stimulus at different angles about the Z-axis. Although it is possible to rotate the stimulus to any degree, the psychometric data only contain human response deviations of 2°, 4°, 8°, and 15° for either direction [18]. The cross-validation and training data were selected from a range of 1° to 20° in deviation for both sides. This range covered all possible degrees of deviation from a soccer goalkeeper’s point of view, considering the distance of the ball from the goal and the physical width of the goal. This kind of scenario is commonly found in a penalty kick situation. 

To validate the model, k-fold cross-validation (k = 5) was used, and the training was carried out within the range of degrees from 7° to 20°. Moreover, the model was tested using the angles obtained from a psychophysical study [16,17,18].

### 3.1. Local Motion Energy and Opponent Motion Neurons

Our model now includes clockwise and counterclockwise detectors in addition to the vertical and horizontal expansion and contraction neurons previously implemented in the first level of local motion energy detectors, as per Casile and Giese [8]. For the second level, we created 20 receptive fields for each clockwise and counterclockwise detection, covering a 36 × 31 local motion detection grid of the lower layer. 

Each rotation receptive field was linked to four contiguous and overlapping subfields, which allowed for the rotation detector to detect the highest rotational activity using a max-pooling strategy. Each subfield comprised a 4 × 5 receptive field arrangement for either clockwise or counterclockwise rotation in the opponent-motion detection level. 

**Figure 2:**
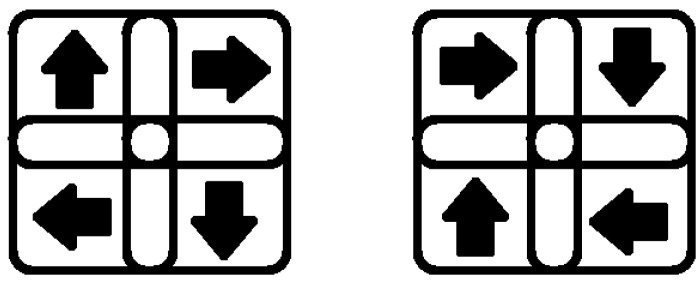
Our model has a receptive field that includes subfields rotating clockwise in one direction.

Here is a clarification of the detection mechanism: Let us take a clockwise rotation receptive field, for example. As mentioned, there are four subfields connected to each detector: the upper-left, the upper-right, the lower-left, and the lower-right. Because our local motion detection level only detects the four fundamental right, left, up, and down directions, the clockwise rotation can only be detected under two conditions depicted in Figure 2. Either of these situations triggers the clockwise rotation detection neuron.

### 3.2. Optic Flow Pattern Neurons

The architecture of the third-level hierarchy remains unchanged from the previous model introduced in Misaghian et al. [16]. To elaborate, there are nine detectors for each direction, and each detector is sensitive to 10 sequential frames out of the 90 frames of the stimulus, summing up to 18 neurons in the optic flow pattern level. For more detailed information, one could refer to [16,17].

### 3.3. Motion Pattern Neurons

Two neurons that recognize complete motion patterns have been assigned to differentiate between leftward and rightward kick stimuli. A robust mutual inhibition model has been used to simulate the interactive dynamics of these decision-making neurons, with the ability to adapt and forget. To solve this nonlinear differential system, we have utilized the 4th order Runge–Kutta method. 

### 3.4. Operating the Simulator

In our research, we designed a forced-choice paradigm task to detect the direction (left or right) of a ball using biological motion stimuli [18]. The task consisted of 960 trials where participants were presented with randomly ordered stimuli of left and right shooting with deviations of 2°, 4°, 8°, and 15° degrees (120 trials for each side and angle). In a previous study, we used a model with three parameters (standard deviation of internal noise, δ; time constant, τ; and inhibitory feedback gain, k) to replicate the psychometric function of 35 athletes, describing their precision in terms of angular deviation [16,17]. In our current study, we have added a new parameter, $\tau_{a}$, which characterizes the starting time point of the adaptation process. This parameter allows for our model to simulate the reaction time of human subjects. Specifically, we calculate the reaction time for each trial by averaging the time points within which the winning decision signal is at its maximum. Our goal is to tune the model to generate the angular threshold and slope of each subject, as well as their average reaction time. To achieve this, we must modify four parameters instead of the previous three:

1. The standard deviation of the added internal noise, *δ*.
2. The time constant, *τ*.
3. The inhibitory feedback gain, *k*, and
4. The time point of adaptation onset, *τ_a_*.

## 4. Results

### 4.1. Extended Model Reaction Time Output

The model has been run for various values of k,τ, and δ, while keeping the value of $\tau_{a}=1.22\;s$ constant. As a result, the angular thresholds, slopes, and reaction times have been calculated. Some of these results are presented in Table 1, which shows how the reaction time changes as all four parameters vary. It is important to note that each reaction time in the psychometric data [18] is the sum of the motor time and the actual reaction time (i.e., the time between stimulus exposure and initiation of the motor response). It is important to note that this study approximated the motor time, which is the time elapsed from initiating a motor response to pressing a button, to be zero. This approximation was made due to the fact that the motor time is only a fraction of the reaction time in our task, with one being in the order of tens of milliseconds [37] and the other being an average of one second. In addition to previous findings, an increase in neurons’ time constant,  τ, not only contributes to smaller angular thresholds and steeper slopes but also decreases reaction time, leading to better performance. On the other hand, an increase in inhibitory gain, *k*, deteriorates and then ameliorates the response accuracy of the model after a specific inhibition gain value, but it always leads to a higher reaction time. The shaded cells in Table 1 show how doubling the inhibitory gain, *k*, results in a lower angular threshold and a steeper slope, while the reaction times of both conditions are unrealistically large, which is expected considering that *k* is too large for both conditions. It is believed that executive function deficit, which is a slower cognitive processing speed, could be a result of disorders such as autism and attention deficit hyperactivity disorder [38,39]. Additionally, some forms of autism are believed to be caused by a high ratio of excitation/inhibition in neuronal systems [40].

It is interesting to note that our model displays accuracy within the expected range despite having a high reaction time, τ, when k is large. This behavior is similar to what is seen in individuals with autism. Our previous research supports this idea, indicating that certain states of our model may be associated with some forms of autism [16,17,38,39,40].

**Figure 3:**
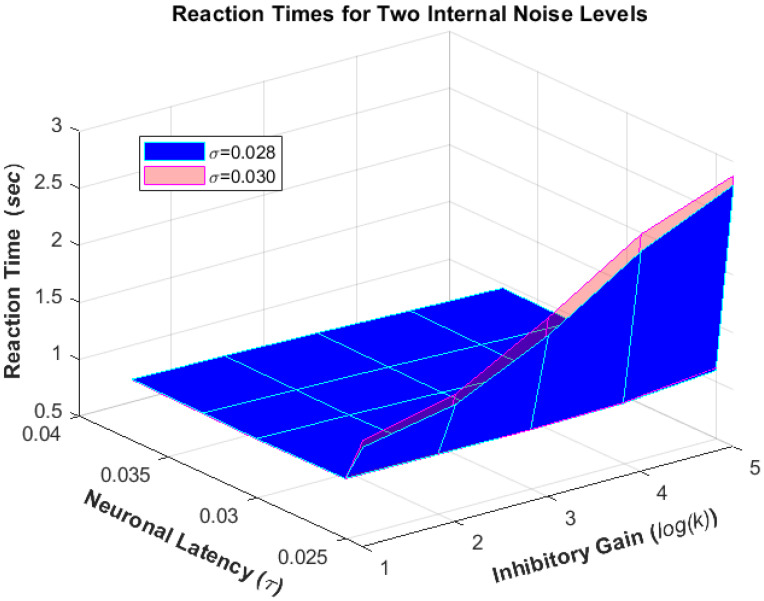
The model was run for various combinations of neuronal latency (τ=0.024, 0.025, 0.03, 0.033, 0.037 s), inhibitory gain (k=2, 4, 8, 16, 32), and noise levels (δ=0.028, 0.030, 0.034) to produce reaction times.

Additionally, when the internal noise level (δ) increases, it results in a higher angular threshold, flatter slope, and faster reaction time (Figure 3). This ultimately leads to worse performance of the simulation model.

Table 1 below shows the simulated observer’s reaction times, corresponding angular thresholds, and slopes for various ranges of τ and k.

### 4.2. Integration of Rotation Detection in Opponent Motion Hierarchy Level

As mentioned earlier, we added rotation detection receptive fields in the opponent-motion hierarchy level to our extended model. This addition has led to better performance in all computed states of the model, including a lower angular threshold and steeper slope. We fed the rotation features along with expansion/contraction features to the third layer and the opponent motion detectors were trained with rotation-included training data. This was performed in the same fashion as reported in Misaghian et al. [16].

By turning on the rotation receptive fields, we were able to simulate the performance of subject B10, which was not achievable in the previous setup [16,17]. This finding leads us to believe that the visual system of different individuals may deploy a different combination of its expansion/contraction and rotation receptive fields when it comes to biological motion perception. In other words, the representation of the neural hierarchies of the visual system could be diverse at different levels, including the opponent motion detection layer [41]. Therefore, we could potentially mimic the two non-simulated subjects by knowing the right combination of operating expansion/contraction and rotation receptive fields. Implementing this capacity for the model to deploy the appropriate combination would be an exciting subject of future work.

### 4.3. Human Results vs. Simulation Results

In Table 2, we have provided the simulated angular thresholds and slopes, as well as the average reaction time and the four parameters that govern the model in those states, compared to the corresponding experimental values. We used a grid-search approach to simulate these groups based on the similarity of subjects’ angular threshold, slopes, and reaction times, similar to our previous work [16,17]. It is important to note that some subjects did not fit into any group and were considered a group with a single member. This grouping approach helped to gain a more generalized understanding of subjects’ overall behavior. While it is possible to identify each subject with a slightly different set of parameters, it can defeat the purpose of generalization.

**Figure 4:**
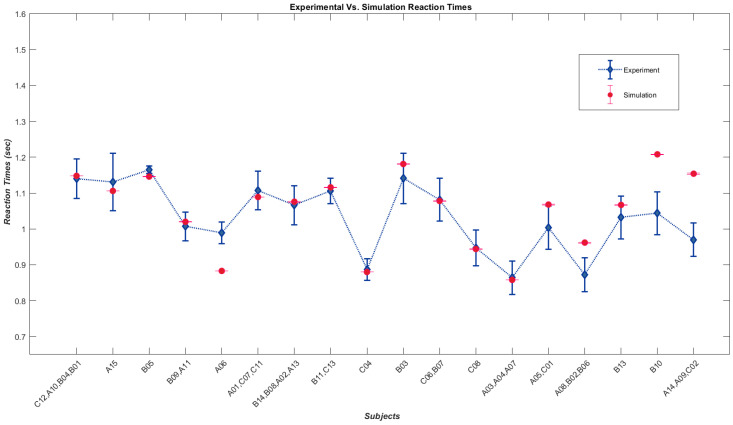
Diamonds represent average reaction times of athlete sub-groups performing psychometric tasks [18], while dots show average reaction times from a model simulating those sub-groups.

Figure 4 shows a plot of the average reaction times of the groups against the corresponding simulated values since the reaction time is the new output feature of our model.

The analysis indicates a significant positive correlation between the experimental and corresponding simulated variables. The Spearman’s correlation coefficient between the experimental and simulated angular thresholds is *r_s_* = 0.984, *p*-value = 2.01 × 10^−27^ (*p* < 0.001). Similarly, the correlation coefficient between the experimental and simulated slope is *r_s_* = 0.955, *p*-value = 1.22 × 10^−19^ (*p* < 0.001). The average reaction time also showed a significant positive correlation between experimental and simulated data, with a correlation coefficient of *r_s_* = 0.513, *p*-value = 0.0014 (*p* < 0.005). The variation in reaction times from subject to subject and our approach to simulating subjects in groups for the sake of generalization is the reason for lower correlation values between experimental and simulated average reaction times. However, if we consider the correlation between simulated average times and the average of each group of subjects, the relationship proves to be strong, with a correlation coefficient of *r_s_* = 0.070, *p*-value = 0.002 (*p* < 0.005).

## 5. Discussion

To achieve better performance by including the rotational optic flow detectors into the mix, obviously, was not a counterintuitive result because, most of the time, more information leads to better decisions. However, the interesting finding was the discovery of a more flexible and broader range of angular threshold-to-slope relationships in psychometric curves that enable the model to encompass a noticeably bigger spectrum of behaviours. There were three subjects out of 38 in the subject pool whose behaviour could not be simulated before [16,17]. The fact that such accuracy behaviours (psychometric functions) exist in the human data is a reliable indicator of a missing factor or factors in the previous setups. Moreover, managing to simulate one of these subjects by including the rotational features postulates that the utilization of such features is essential, but that they most likely differ from individual to individual. Unfortunately, our simulation model could not estimate the extent of it. 

Furthermore, empowering the model to have a reaction time using our physiologically plausible strategy not only seemed necessary for a thorough simulation model but also appeared to be a source of new information and disambiguation. For instance, the earlier setup could achieve similar behaviours with different tunings, and it was only possible to pick the proper set of parameters by examining the characteristics of the decision signals. However, in the extended model, the reaction time showed which tuning complies with human behavior. Also, as mentioned in the Section 4, the same accuracy behavior but with a much longer reaction time could be deemed as a result of executive function deficit, and analyzing the model’s parameters showed that such states occur when mutual inhibition is high. Interestingly, it has been suggested that some forms of autism could be the result of an increased ratio of excitation/inhibition in sensory, mnemonic, and some other systems [40]. 

Numerous limitations to the present model exist. For example, the opponent-motion neuron in the model only receives signals from adjoining receptive fields, and there is no detector to pool the signals from two distant receptive fields. Hence, it is unable to incorporate any global relative motion in a moving scene [19]. Also, both the first and second layers were presumed to be noise-free, and no process noise was introduced to the model. This is going to be considered in the future work.

Furthermore, the feed-forward assumption in our model imposes fixed prototypes, parameters, and priors, which apparently is far from what happens in the human visual system. A more inclusive model must also be capable of learning and updating. Therefore, the integration of such capacities lies in our future work.

Finally, integrating a descriptive-level model of mirror neurons into the biological motion perception model is critical. A mirror neuron is a type of neuron that fires during both action execution and action observation [42]. This particular feature has been the source of speculation that these cells are the neural substrate of understanding the intentions of others through observation [43,44]. Moreover, there is some overlap between biological motion perception areas and areas where mirror neurons are believed to reside [45,46,47]. Consequently, investigation of the role of mirror neurons in action prediction from biological motion becomes crucially important. Therefore, an augmented biological motion perception model, including a descriptive model of mirror neurons, can be a great asset not only for the investigation of biological motion perception but also for understanding phenomena like motor imagery (MI), which is the mental simulation of movement without physical execution. It was shown that a training platform using MI to control an EEG-based brain–computer interface could help teach MI skills in healthy and post-stroke groups [48]. As mentioned above, an augmented model can shed light on the underlying mechanisms and help to develop more effective rehabilitation methods like the above. Ultimately, a descriptive model of biological motion perception that includes a mirror neuron system is one of the highest priorities for future work.

## Figures and Tables

**Table 1:** The psychometric function’s slope and angular threshold, along with the average reaction time of the model, were computed for different values of τ and k, at two different noise levels (δ=0.030 and δ=0.034) when $\tau_{a}$ was set to 1.22. These results have been reported below.

	Threshold			*τ*				Threshold			*τ*		
	δ=0.030	0.024	0.025	0.03	0.033	0.037		δ=0.034	0.024	0.025	0.03	0.033	0.037
	2	9.82	5.57	5.28	5.20	5.02		2	12.26	6.40	5.89	5.81	5.67
*k*	4	10.96	5.96	5.60	5.59	5.43	*k*	4	13.97	6.87	6.41	6.17	5.98
	8	13.97	6.83	6.34	6.15	5.93		8	16.32	7.73	7.23	7.05	6.80
	16	14.72	8.30	7.69	7.40	6.96		16	16.71	9.28	8.59	8.47	7.95
	32	14.88	10.18	9.31	9.04	8.91		32	15.45	11.13	10.59	10.26	9.90
	Slope			*τ*				Slope			*τ*		
	δ=0.030	0.024	0.025	0.03	0.033	0.037		δ=0.034	0.024	0.025	0.03	0.033	0.037
	2	0.10	0.23	0.26	0.28	0.29		2	0.08	0.19	0.22	0.24	0.24
*k*	4	0.10	0.22	0.26	0.25	0.28	*k*	4	0.08	0.18	0.21	0.22	0.24
	8	0.08	0.20	0.22	0.24	0.27		8	0.07	0.16	0.19	0.20	0.22
	16	0.09	0.18	0.20	0.21	0.24		16	0.07	0.15	0.17	0.17	0.20
	32	0.10	0.16	0.18	0.19	0.19		32	0.09	0.14	0.15	0.16	0.16
	RT			*τ*				RT			*τ*		
	δ=0.030	0.024	0.025	0.03	0.033	0.037		δ=0.034	0.024	0.025	0.03	0.033	0.037
	2	1.375	1.028	1.019	1.030	1.039		2	1.434	1.025	1.014	1.022	1.034
*k*	4	1.530	1.021	1.006	1.014	1.025	*k*	4	1.622	1.019	0.996	1.007	1.019
	8	1.915	1.016	0.985	0.994	1.006		8	2.070	1.017	0.976	0.986	0.996
	16	2.433	1.027	0.957	0.968	0.981		16	2.581	1.033	0.948	0.960	0.974
	32	2.793	1.096	0.938	0.948	0.960		32	2.876	1.121	0.928	0.937	0.951

**Table 2:** Simulating athletes’ psychometric functions by adjusting k, τ, δ, and $\tau_{a}$ to control angular threshold (75%) and slope, along with average reaction times.

Rotation Detectors Are Active for Simulating B10										
Subjects	Angular Thresholds from Experiment	Angular Thresholds from Simulation	Slopes from Experiment	Slopes from Simulation	Reaction Time from Experiment	Reaction Time from Simulation	Inhibitory Gain (*k*)	Time Constant (τ)	Noise ( δ )	Adaptation Onset ($\tau_{a}$)
**‘C12’**	4.041 ± 1.06	**5.252 ± 0.20**	0.261 ± 0.030	**0.263 ± 0.0049**	0.994 ± 0.07	**1.148 ± 0.0005**	4	0.025	0.022	1.22
**‘A10’**	4.176 ± 1.08	**˶**	0.252 ± 0.029	**˶**	0.929 ± 0.04	**˶**	˶	˶	˶	˶
**‘B04’**	4.506 ± 1.11	**˶**	0.246 ± 0.028	**˶**	1.194 ± 0.05	**˶**	˶	˶	˶	˶
**‘B01’**	4.805 ± 1.13	**˶**	0.243 ± 0.027	**˶**	1.443 ± 0.06	**˶**	˶	˶	˶	˶
**‘A15’**	5.321 ± 1.15	**5.317 ± 0.19**	0.242 ± 0.025	**0.276 ± 0.005**	1.131 ± 0.08	**1.106 ± 0.0002**	2	0.033	0.032	1.34
**‘B05’**	5.361 ± 1.05	**5.201 ± 0.18**	0.284 ± 0.028	**0.307 ± 0.0055**	1.165 ± 0.01	**1.146 ± 0.0002**	4	0.037	0.030	1.40
**‘B09’**	6.602 ± 1.42	**6.872 ± 0.27**	0.188 ± 0.021	**0.180 ± 0.0036**	1.001 ± 0.03	**1.020 ± 0.0003**	4	0.025	0.034	1.22
**‘A11’**	6.637 ± 1.52	**˶**	0.171 ± 0.023	**˶**	1.013 ± 0.05	**˶**	˶	˶	˶	˶
**‘A06’**	6.609 ± 1.22	**6.793 ± 0.25**	0.233 ± 0.020	**0.200 ± 0.0038**	0.989 ± 0.03	**0.883 ± 0.0002**	8	0.033	0.032	1.10
**‘A01’**	7.000 ± 1.52	**6.909 ± 0.25**	0.175 ± 0.02	**0.205 ± 0.0038**	1.007 ± 0.01	**1.089 ± 0.0003**	8	0.030	0.034	1.40
**‘C07’**	7.097 ± 1.42	**˶**	0.192 ± 0.02	**˶**	1.169 ± 0.07	**˶**	˶	˶	˶	˶
**‘C11’**	7.165 ± 1.39	**˶**	0.197 ± 0.02	**˶**	1.146 ± 0.08	**˶**	˶	˶	˶	˶
**‘B14’**	7.692 ± 1.80	**7.701 ± 0.36**	0.147 ± 0.018	**0.131 ± 0.0032**	1.005 ± 0.04	**1.076 ± 0.0009**	1	0.024	0.026	0.96
**‘B08’**	7.753 ± 1.81	**˶**	0.146 ± 0.018	**˶**	0.923 ± 0.06	**˶**	˶	˶	˶	˶
**‘A02’**	7.837 ± 1.87	**˶**	0.141 ± 0.018	**˶**	1.133 ± 0.04	**˶**	˶	˶	˶	˶
**‘A13’**	7.873 ± 1.69	**˶**	0.159 ± 0.018	**˶**	1.203 ± 0.08	**˶**	˶	˶	˶	˶
**‘B11’**	8.132 ± 2.00	**8.459 ± 0.39**	0.132 ± 0.017	**0.124 ± 0.0031**	1.065 ± 0.02	**1.116 ± 0.0009**	1	0.024	0.028	1.00
**‘C13’**	8.594 ± 2.09	**˶**	0.128 ± 0.017	**˶**	1.147 ± 0.05	**˶**	˶	˶	˶	˶
**‘C04’**	9.173 ± 1.78	**9.685 ± 0.35**	0.158 ± 0.017	**0.148 ± 0.003**	0.887 ± 0.03	**0.880 ± 0.0003**	16	0.025	0.034	1.04
**‘B03’**	9.191 ± 2.64	**9.292 ± 0.45**	0.103 ± 0.016	**0.111 ± 0.0029**	1.141 ± 0.07	**1.181 ± 0.0012**	2	0.024	0.028	1.00
**‘C06’**	9.543 ± 2.34	**9.709 ± 0.41**	0.118 ± 0.016	**0.123 ± 0.0029**	0.899 ± 0.05	**1.078 ± 0.001**	2	0.024	0.030	0.90
**‘B07’**	9.589 ± 2.86	**˶**	0.096 ± 0.016	**˶**	1.264 ± 0.07	**˶**	˶	˶	˶	˶
**‘C08’**	9.747 ± 1.70	**9.838 ± 0.32**	0.170 ± 0.017	**0.167 ± 0.0031**	0.947 ± 0.05	**0.944 ± 0.0003**	32	0.033	0.032	1.22
**‘A03’**	10.490 ± 1.56	**12.076 ± 0.43**	0.130 ± 0.011	**0.130 ± 0.0028**	0.964 ± 0.04	**0.858 ± 0.0006**	32	0.025	0.340	0.88
**‘A04’**	10.801 ± 2.20	**˶**	0.132 ± 0.016	**˶**	0.757 ± 0.05	**˶**	˶	˶	˶	˶
**‘A07’**	10.843 ± 2.26	**˶**	0.128 ± 0.016	**˶**	0.871 ± 0.05	**˶**	˶	˶	˶	˶
**‘A05’**	10.770 ± 2.72	**10.747 ± 0.41**	0.105 ± 0.015	**0.130 ± 0.0029**	1.098 ± 0.06	**1.068 ± 0.0014**	4	0.024	0.028	0.80
**‘C01’**	10.830 ± 2.61	**˶**	0.110 ± 0.016	**˶**	0.909 ± 0.06	**˶**	˶	˶	˶	˶
**‘A08’**	12.132 ± 2.76	**12.722 ± 0.45**	0.109 ± 0.015	**0.124 ± 0.0027**	0.793 ± 0.03	**0.962 ± 0.0013**	4	0.024	0.032	0.66
**‘B02’**	12.173 ± 2.68	**˶**	0.113 ± 0.015	**˶**	0.936 ± 0.05	**˶**	˶	˶	˶	˶
**‘B06’**	12.525 ± 2.82	**˶**	0.108 ± 0.015	**˶**	0.888 ± 0.06	**˶**	˶	˶	˶	˶
**‘B13’**	12.860 ± 3.94	**11.549 ± 0.49**	0.078 ± 0.015	**0.109 ± 0.0028**	1.032 ± 0.06	**1.067 ± 0.0011**	2	0.024	0.034	0.84
*’B10’	13.160 ± 3.02	**13.363 ± 0.56**	0.103 ± 0.015	**0.101 ± 0.0027**	1.044 ± 0.06	**1.208 ± 0.0009**	64	0.025	0.036	1.15
**‘A14’**	16.617 ± 4.89	**17.319 ± 0.74**	0.071 ± 0.014	**0.088 ± 0.0025**	1.058 ± 0.06	**1.154 ± 0.0017**	4	0.024	0.038	0.60
**‘A09’**	17.194 ± 5.84	**˶**	0.061 ± 0.014	**˶**	1.014 ± 0.04	**˶**	˶	˶	˶	˶
**‘C02’**	17.787 ± 5.36	**˶**	0.068 ± 0.014	**˶**	0.842 ± 0.04	**˶**	˶	˶	˶	˶

“˶” indicates that the simulated values are the same for the subjects listed below the subject with actual value.

## Data Availability

Data are contained within the article.
